# Pediatric dermatofibrosarcoma protuberans: A clinicopathologic and genetic analysis of 66 cases in the largest institution in Southwest China

**DOI:** 10.3389/fonc.2023.1017154

**Published:** 2023-01-27

**Authors:** Zhang Zhang, Yang Lu, Changle Shi, Min Chen, Xin He, Hongying Zhang

**Affiliations:** Department of Pathology, West China Hospital, Sichuan University, Chengdu, China

**Keywords:** dermatofibrosarcoma protuberans, giant cell fibroblastoma, pediatric sarcoma, COL1A1-PDGFB fusion, COL3A1-PDGFB fusion, fluorescence *in situ* hybridization, next generation sequencing

## Abstract

**Background:**

Dermatofibrosarcoma protuberans (DFSP) is an uncommon cutaneous tumor in children. Most published articles are sporadic or small series and lack systematically molecular analyses. The aim of our study is to better understand the clinicopathologic and genetic features of these rare lesions.

**Methods:**

All patients diagnosed with DFSP aged ≤ 18 years were retrospectively reviewed from January 2006 to May 2022.

**Results:**

A total of 66 cases (32 male and 34 female patients) were identified, with ages ranging from 0.3 to 18 years (median, 13 years). Tumor locations predominantly occurred on the trunk (38/66, 57.6%), followed by the extremities (20/66, 30.3%) and head/neck (8/66, 12.1%). Histological findings revealed classic (41/66, 62.1%), myxoid (4/66, 6.1%), pigmented (6/66, 9.1%), plaque-like (3/66, 4.5%), giant cell fibroblastoma (GCF; 6/66, 9.1%), and fibrosarcomatous (6/66, 9.1%) variants of DFSP. Immunochemistry revealed minority tumors (9/66, 13.6%) showing patchy or negative staining for CD34. Fluorescence *in situ* hybridization (FISH) indicated that 49 of 53 tested cases including all detected biopsy specimens (11/11) contained *COL1A1*-*PDGFB* fusion, in which the average copy number gain of *COL1A1*-*PDGFB* was 0.68. There were four cases negative for *COL1A1-PDGFB* rearrangement, one of which was found to harbor a novel *COL3A1*-*PDGFB* fusion by next-generation sequencing (NGS). Treatment for 63 patients comprised 40 marginal excisions and 23 wide local excisions (WLEs), including 1 with imatinib therapy. Follow-up information was available on 49 patients with a duration of 12–161 months (median, 60 months). Fourteen patients developed tumor recurrence, all with initial marginal excisions. The others survived with no evidence of disease.

**Conclusions:**

This study of pediatric DFSP indicates certain discrepancies in clinicopathologic characteristics between children and adults. The majority of pediatric DFSPs contain *COL1A1*-*PDGFB* fusion, the same as their adult counterparts. The *COL3A1*-*PDGFB* chimerism might be associated with the special morphology of GCF, which needs further investigation. FISH is valuable in biopsy tissues and cases with atypical CD34 immunostaining, while supplementary NGS could be helpful to identify the cytogenetically cryptic DFSP. Overall, an urgent accurate diagnosis is needed to formulate an optimal therapeutic strategy in the pediatric population.

## Introduction

Dermatofibrosarcoma protuberans (DFSP), one of the most common dermal sarcomas, is a locally infiltrative dermal and subcutaneous fibroblastic tumor of intermediate malignancy (1). According to the 2020 World Health Organization (WHO) classification of soft tissue and bone tumors, the lesion divides into several histologic subtypes, including classic DFSP, pigmented DFSP, myxoid DFSP, DFSP with myoid differentiation, plaque-like DFSP, giant cell fibroblastoma (GCF), and fibrosarcomatous DFSP (FS-DFSP) (1). DFSP can locally recur with a rate of 20%–50%, of which FS-DFSP is the only subtype associated with more aggressive behavior. Approximately 10%–16% of FS-DFSP may develop distant metastases, resulting in a worse prognosis (1–4).

Cytogenetically, more than 90% of DFSP cases are characterized with the collagen type I alpha 1 and platelet-derived growth factor B (*COL1A1-PDGFB*) fusion deriving from supernumerary ring r (17;22) or translocation t (17;22) (q22; q13) (5). The chimeric formation could result in upregulating the PDGFB expression, associated with tumorigenesis and accordingly designed to be the target by tyrosine kinase inhibitors like imatinib mesylate (6). In clinical routine practice, the critical molecular abnormality is exploited to be detected by fluorescence *in situ* hybridization (FISH), aiding in diagnosis and validating targeted molecular therapy in certain circumstances (7, 8).

The incidence of DFSP is approximately 1 case per 1,000,000 person-years of adults predominantly within the second to fifth decades, while even rare in children (9–12). Notably, to the best of our knowledge, previous reports are mostly sporadic or small series and there are only six large analyses involving pediatric DFSP, most of which are absent of detailed histological analysis or systematic molecular studies (11, 13–17). Therefore, we retrospectively evaluated a large series of 66 pediatric DFSPs at the largest institution in Southwest China and aimed to further investigate the clinicopathological features and molecular characteristics.

## Materials and methods

### Patient acquisition

This study was approved by the West China Hospital Institutional Review Board. A SNOMED search of the hospital surgical pathology and consultation files from January 2006 to May 2022 identified 926 DFSPs. All cases were independently reviewed by two pathologists (HZ and ZZ) who specialized in soft tissue tumors and two general surgical pathologists (XH and YL). The patients diagnosed with DFSP aged ≥ 19 years were considered adult DFSP and were excluded. Finally, 66 DFSP cases aged ≤ 18 years were confirmed as pediatric DFSP and included in this study.

### Immunohistochemical staining

Immunohistochemical analysis was performed using the EnVision Plus detection system (DAKO, Carpinteria, CA, USA) with positive and negative controls. Antibodies for the experiments included CD34 (EP88, ready-to-use; ZSGB-Bio), SMA (UMAB237, ready-to-use; ZSGB-Bio), Ki-67 (clone MIB-1, 1:100; Dako), desmin (D33, 1:100; Dako), S-100 protein (4C49, 1:100; Dako), myogenin (F5D, 1:50; ZSGB-Bio), Bcl-2 (EP36, 1:200; ZSGB-Bio), CD99 (EP8, ready-to-use; ZSGB-Bio), p16 (16P04/IC2, ready-to-use; Dako), p63 (4A4, 1:400; Dako), cytokeratin (AE1/AE3, 1:100; Dako), and EMA (E29, 1:100; Dako).

### Fluorescence *in situ* hybridization

FISH analyses were performed using commercially available ZytoLight^®^ SPEC *PDGFB* Dual Color Break Apart Probe and ZytoLight^®^ SPEC *COL1A1*/*PDGFB* Dual Color Dual Fusion Probe (ZytoVision, Bremerhaven, Germany). The tests were performed on 4-μm sections according to an established laboratory protocol, as previously described (18). Two investigators independently counted at least 100 nuclei on each slide. A case was considered positive for *PDGFB* rearrangement when at least ≥10% of the cells exhibited a split signal pattern which showed that the distance between the green and red signals was greater than the diameter of two signals. The *COL1A1*-*PDGFB* fusion was considered positive when at least 10% of the cells showed one separate red signal, one separate green signal, and two red/green fusion signals. The *COL1A1*-*PDGFB* copy gain was calculated according to the description of Abbott et al. (19).

### Next-generation sequencing

Genomic profiling was performed on FFPE tissues with capture-based panel targeting 481 soft tissue tumor-relevant genes. Genomic DNA was extracted from FFPE tissues using a QIAamp DNA FFPE Tissue Kit (Qiagen, Hilden, Germany) according to the manufacturer’s protocol. The eligible DNA was hybridized with the capture probes, selected using magnetic beads, and polymerase chain reaction (PCR)-amplified. Then, library fragment size was determined by Bioanalyzer2100 (Agilent Technologies, Santa Clara, CA). The target-enriched library was sequenced on the Hiseq4000 platform (Illumina, Inc., San Diego, CA) following the manufacturer’s instructions. The average sequencing depth was 1000× for all targeted regions.

### Polymerase chain reaction and Sanger sequencing

The *COL3A1*-*PDGFB* fusion was analyzed by PCR using primers (COL3A1-F: 5’-CTTCAGGGTGAGACAGCCAA-3’; PDGFB-R: 5’-CATAAGCCCCCGGATTTGGT-3’). Sanger sequencing was performed at Tsingke Biological Technology Co., Ltd. (Chengdu, China).

### Statistical analysis

Statistical analysis was performed using the GraphPad Prism version 5 (GraphPad Software, San Diego, CA). Comparisons between different groups were evaluated using Student’s *t*-test and ANOVA for continuous variables. *p* < 0.05 indicates the statistical significance between different groups.

## Results

### Clinicopathological features of the study cohort

The relevant clinicopathologic data are summarized in **Table 1**. The study cohort comprised 32 male and 34 female patients (ratio, 1:1.1). The age of the patients ranged from 0.3 to 18 years (median, 13 years; mean, 11.38 years), including 2 infants (2/66, 3.0%; age ≤ 11 months) and 26 children aged less than 10 years (26/66, 39.4%). The tumor size ranged from 0.6 to 8 cm (median, 3 cm; mean, 2.9 cm). The majority of the tumors occurred on the trunk (38/66, 57.6%), followed by the extremities (20/66, 30.3%) and head/neck (8/66, 12.1%). The clinical manifestations commonly presented as nodular or multinodular masses (61/66, 92.4%) and much less in the plaque stage (5/66, 7.6%). The cut surfaces of DFSP were tan and yellow, with rubbery firm to soft gelatinous appearances. There were 15 cases (15/66, 22.7%) who underwent biopsies before treatment.

**Table 1:** Clinicopathological and cytogenetic features of 66 pediatric DFSPs.

No.	Age of operation(years)	Gender	Location	Size (cm)	Primary subtypes	Recurrent subtypes	CD34	SMA	PDGFB break signal	COLA1-PDGFB fusion signal	Surgery	Local recurrence (months)	Metastasis	Died	Follow-up (months)
1	16	F	Abdomen	3.0	Classic	FS	Negative	NA	Positive	Positive	E	40	No	No	NED/115
2	17	F	Shoulder	3.5	FS	FS	Positive	NA	Positive	Positive	E	20	NA	NA	NA
3	15	M	Chest	6.0	Classic	FS	Positive	NA	Positive	Positive	E	55	No	No	NED/114
4	6	M	Chest	2.0	Classic	NA	Positive	NA	Positive	Positive	WE	No	No	No	NED/94
5	16	F	Chest	2.2	Classic	Classic	Positive	NA	Failure	Positive	E	10	No	No	NED/149
6	17	M	Abdomen	2.5	Classic	NA	Positive	Negative	Positive	Positive	E	NA	NA	NA	NA
7	8	M	Shoulder	3.0	GCF	GCF	Positive	Negative	Positive	Positive	E	9, 34	No	No	NED/161
8	15	F	Chest	3.5	Classic	NA	Positive	NA	Positive	Positive	WE	No	No	No	NED/137
9	17	F	Abdomen	1.3	Classic	NA	Positive	NA	Positive	Positive	NA	No	No	No	NED/66
10	12	M	Chest	2.2	Myxoid	NA	Positive	NA	Positive	Positive	WE	No	No	No	NED/74
11	10	M	Chest	5.2	Classic	Classic	Positive	Negative	Positive	Positive	E	13	No	No	NED/81
12	15	F	Abdomen	3.5	Classic	Classic	Positive	Negative	Positive	Positive	E	22	No	No	NED/85
13	17	F	Chest	3.0	Classic	NA	Positive	NA	Failure	Positive	WE	NA	NA	NA	NA
14	4	F	Finger	2.5	Pigmented	Pigmented	Positive	Negative	Positive	Positive	E	5	No	No	NED/61
15	11	M	Forearm	0.7	FS	NA	Positive	Negative	Positive	Positive	WE	No	No	No	NED/60
16	0.3	F	Shoulder	2.8	Myxoid	NA	Patchy	Focal	Negative	Positive	E	No	No	No	NA
17	13	F	Chest	1.2	Classic	NA	Positive	Focal	Positive	Positive	WE	No	No	No	NED/101
18	6	M	Thigh	1.6	Classic	NA	patchy	Negative	Positive	Positive	WE	No	No	No	NED/100
19	3	M	Neck	4.0	GCF	NA	Positive	Negative	Positive	Positive	E	60	No	No	NED/89
20	11	M	Foot	2.0	Pigmented	NA	Positive	Negative	Positive	Positive	NA	No	No	No	NA
21	14	F	Scalp	2.5	Classic	NA	Positive	Negative	Positive	Positive	WE	No	No	No	NED/75
22	18	F	Back	1.8	Classic	NA	Positive	Negative	Positive	Positive	E	No	No	No	NA
23	16	M	Back	1.5	Myxoid	Myxoid	Patchy	Negative	Negative	Negative	E	10	No	No	NED/77
24	18	F	Face	3.0	Plaque-like	NA	Positive	Negative	Positive	Positive	E	No	No	No	NA
25	17	F	Upper arm	4.0	Classic	NA	Positive	Negative	Positive	Positive	WE	No	No	No	NA
26	17	F	Chest	1.0	Classic	NA	Positive	Negative	Positive	Positive	E	No	No	No	NA
27	7	M	Leg	1.0	Classic	NA	Positive	Negative	NA	NA	E	No	No	No	NED/82
28	17	M	Back	5.0	Classic	NA	patchy	Negative	NA	NA	E	No	No	No	NED/79
29	18	M	Head	2.5	Classic	NA	Positive	Focal	NA	NA	E	No	No	No	NED/73
30	4	F	Back	3.0	Classic	NA	Positive	Negative	NA	NA	WE	No	No	No	NED/15
31	17	M	Thigh	NA	Classic	NA	Positive	Negative	Positive	Positive	WE	No	No	No	NED/14
32	17	M	Abdomen	3.5	Classic	NA	Positive	Focal	NA	NA	E	No	No	No	NED/69
33	0.9	M	Chest	4.0	GCF	NA	Positive	Negative	Positive	Positive	WE	No	No	No	NED/59
34	9	M	Thigh	NA	Classic	NA	Positive	Negative	NA	NA	E	No	No	No	NED/58
35	9	F	Waist	NA	Classic	NA	Positive	Negative	NA	NA	E	No	No	No	NED/29
36	8	F	Abdomen	NA	Classic	NA	Positive	Negative	NA	NA	E	No	No	No	NED/50
37	7	F	Abdomen	NA	Classic	NA	Positive	Negative	Positive	Positive	E	No	No	No	NED/26
38	6	M	Chest	NA	Classic	NA	Positive	Negative	Positive	Positive	WE	No	No	No	NED/9
39	5	M	Leg	NA	Classic	NA	Positive	Focal	Positive	Positive	WE	No	No	No	NA
40	4	M	Neck	2.8	Myxoid	NA	Patchy	NA	Positive	Positive	E	8	No	No	NED/27
41	4	F	Forearm	3.0	Pigmented	NA	Positive	Negative	Positive	Positive	E	16	No	No	NED/45
42	1	F	Hip	4.0	Pigmented	NA	Positive	Negative	Negative	Negative	E	No	No	No	NED/44
43	18	M	Abdomen	NA	Classic	NA	Positive	NA	Positive	Positive	E	No	No	No	NED/44
44	18	F	Abdomen	NA	Classic	NA	Positive	Negative	NA	NA	E	12, 12, 48	No	No	NED/66
45	18	F	Groin	NA	FS	NA	Patchy	Negative	NA	NA	WE	No	No	No	NED/48
46	15	M	Axilla	3.0	Classic	NA	Positive	Negative	Positive	Positive	WE	No	No	No	NED/26
47	14	F	Breast	NA	Classic	NA	Positive	NA	NA	NA	NA	No	No	No	NA
48	14	F	Neck	NA	Classic	NA	Positive	NA	Positive	Positive	WE	No	No	No	NED/54
49	13	F	Abdomen	NA	Classic	NA	Positive	Negative	NA	NA	WE	No	No	No	NED/26
50	13	M	Back	8.0	Classic	NA	Positive	NA	Positive	Positive	WE	No	No	No	NED/34
51	12	M	Shoulder	NA	Pigmented	NA	Positive	NA	NA	NA	E	No	No	No	NED/23
52	13	F	Forehead	NA	Classic	NA	Negative	NA	Negative	Negative	E	No	No	No	NA
53	18	F	Breast	NA	Classic	NA	Positive	NA	Positive	Positive	WE	No	No	No	NED/61
54	3	M	Scalp	NA	Classic	NA	Positive	Negative	Positive	NA	E	No	No	No	NED/23
55	15	F	Shoulder	NA	Classic	NA	Positive	Negative	Positive	Positive	E	No	No	No	NED/22
56	6	M	Abdomen	NA	Classic	NA	Positive	Negative	Positive	Positive	WE	No	No	No	NED/21
57	5	F	Sacrococcygeal region	3.0	GCF	NA	Positive	Positive	Positive	Positive	E	No	No	No	NA
58	11	M	Forearm	3.0	NA	Pigmented	Positive	Focal	Positive	Positive	E	72	No	No	NED/84
59	18	F	Breast	0.6	Classic	NA	Positive	NA	Positive	Positive	E	No	No	No	NED/12
60	11	M	Thigh	NA	FS	NA	Positive	NA	Positive	Positive	E	72	No	No	NED/88
61	9	F	Breast	NA	Plaque-like	NA	Positive	Focal	Positive	Positive	E	No	No	No	NED/57
62	17	F	Abdomen	NA	Classic	NA	Positive	NA	Positive	Positive	WE	No	No	No	NED/12
63	2	M	Groin	3.0	GCF	NA	Positive	Negative	Positive	Positive	E	No	No	No	NED/25
64	10	M	Waist	NA	Plaque-like	NA	Positive	Negative	Positive	Positive	WE	No	No	No	NED/2
65	2	M	Forearm	NA	GCF	NA	Patchy	NA	Ambiguous	Negative	E	No	No	No	2
66	13	F	Groin	NA	Classic	NA	Positive	Negative	Positive	Positive	E	No	No	No	1

FS, fibrosarcomatous; GCF, giant cell fibroblastoma; NA, not available; E, excision; WE, wide excision; NED, no evidence of disease.

### Histologic features

The tumors were classified according to histologic subtypes into classic DFSP (41/66, 62.1%), myxoid DFSP (4/66, 6.1%), pigmented DFSP (6/66, 9.1%), plaque-like DFSP (3/66, 4.5%), giant cell fibroblastoma (GCF; 6/66, 9.1%), and fibrosarcomatous DFSP (6/66, 9.1%).

**Figure 1:**
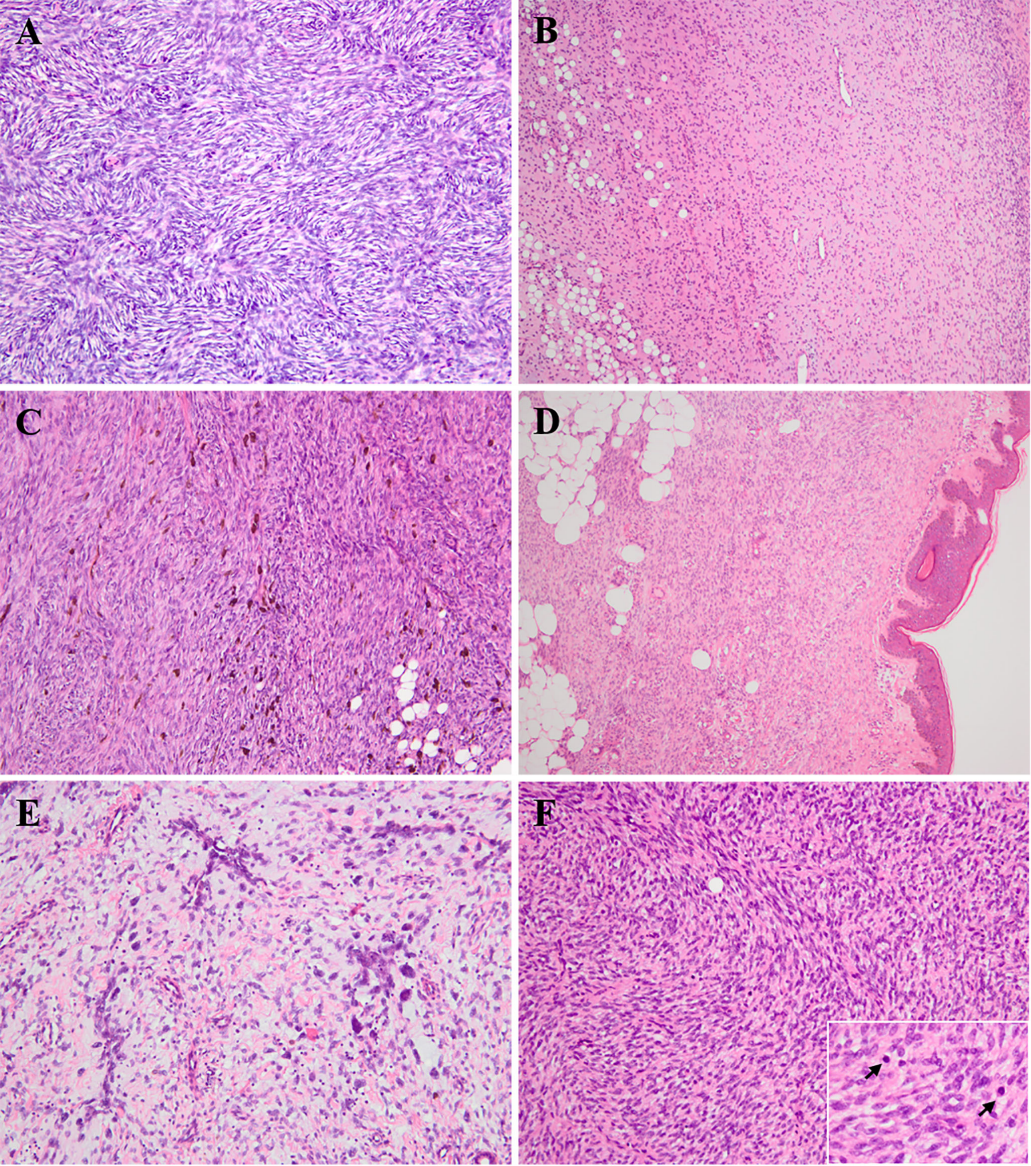
Histologic findings of various subtypes of pediatric DFSP. **(A)** Conventional DFSP. The lesion consisted of uniform bland spindled neoplastic cells arranged in a classic cartwheel pattern (H&E × 200). **(B)** Myxoid DFSP. The tumor had relatively low cellularity and consisted of pump spindle or stellated tumor cells diffusely distributed in prominent myxoid architecture with numerous vessels (H&E × 100). **(C)** Pigmented DFSP. The dendritic cells with melanin pigment punctuated within monotonous storiform area of conventional DFSP (H&E × 200). **(D)** Plaque-like DFSP. The dermal-based lesion composed of regular plump tumor cells presenting a horizontally oriented arrangement and a focal storiform structure (H&E × 100). **(E)** Giant cell fibroblastoma (case 65). High proportion of hyperchromatic multinucleated giant cells scattered in the loosely fibrous and myxoid stroma, and lined pseudovascular spaces (H&E × 200). **(F)** Fibrosarcomatous DFSP. The lesion was composed of high-grade fibrosarcoma-like component, showing a typical herringbone appearance with increasing mitoses (H&E × 200; insert × 400).

Microscopically, the DFSPs diffusely infiltrated into the dermis and subcutis with ill-defined borders, which, with infiltration into the subcutaneous fat, could result in a honeycomb-like appearance. Typically, uniform wavy or spindle tumor cells were proliferative and arranged in a storiform or cartwheel pattern (**Figure 1A**). In the myxoid subtype (cases 10, 16, 23, and 40), the abundant myxoid stroma with low cellularity and numerous vessels occupied more than 50% of the tumor (**Figure 1B**). There were six pigmented-subtype tumors (including cases 14, 20, 41, 42, 51, and 58; case 58 was a recurrent lesion), all of which could find that the pigmented dendritic cells were scattered over fibroblastic tumor cells (**Figure 1C**). The three cases of plaque-like DFSP (cases 24, 61, and 64) were dermal-based lesions, composed of regular plump tumor cells presenting a horizontally oriented arrangement and a focal storiform structure (**Figure 1D**). In six GCF tumors, a varying number of pleomorphic mononucleated or multinucleated giant cells admixed with spindle cells in the loose myxoid matrix or abundant collagenous stroma. Among them, three were pure GCF (cases 19, 33, and 65) and three were hybrid lesions (cases 7, 57, and 63) that consisted of conventional DFSP and GCF components. Most differently, case 65 uniquely consisted of a higher proportion of neoplasms that were with larger and more atypical giant nuclei compared to that of the other five typical GCFs, which predominantly contained slender wavy spindled cells and sporadic giant cells. The floret-like giant cell-lined pseudovascular spaces and infiltrated subcutaneous fat mimicking liposarcoma were easy to find (**Figure 1E**). Most of the above variants of DFSP presented low mitotic activity (0–5/10 high-power fields), while one conventional DFSP showed mitotic activity with 11/10 high-power fields. FS-DFSP presented in four primary cases (cases 2, 15, 45, and 60) and two recurrent lesions (cases 1 and 3), whose primary tumors were both confirmed as a conventional DFSP. The fibrosarcomatous component was composed of neoplastic cells with increased cellularity and mitotic activity, arranging in a fascicular pattern with a herringbone appearance (**Figure 1F**). Necrosis was not identified.

### Immunohistochemistry analysis

**Figure 2:**
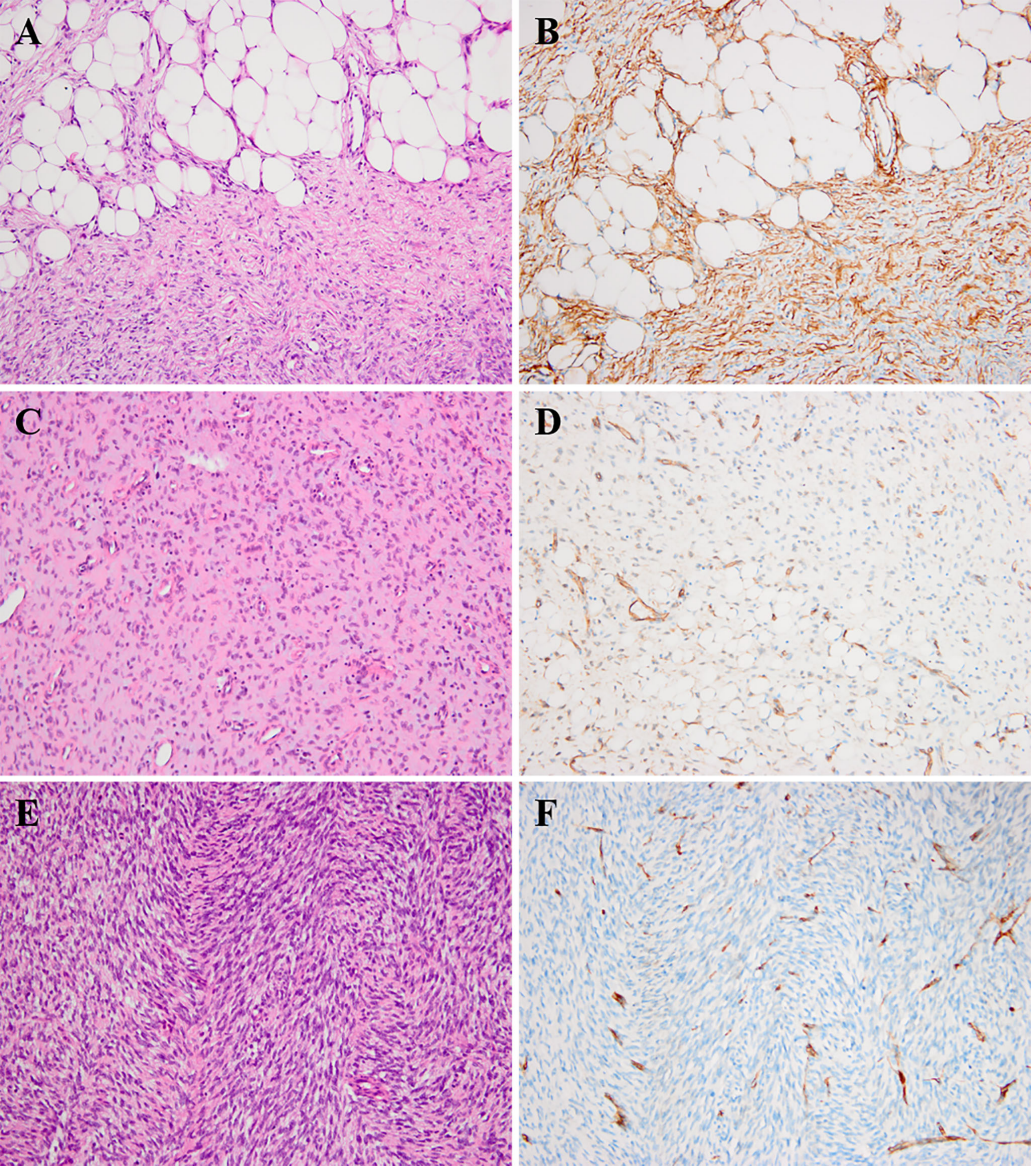
Histologic findings of DFSP and corresponding immunohistochemical images. **(A, B)** The neoplastic cells infiltrated into subcutaneous fat tissues forming a characteristic “honeycomb” pattern (**A,** H&E × 200). The corresponding component showed diffuse positivity for CD34 (**B,** × 200). **(C, D)** The myxoid component of DFSP (case 40) could show patchy or focal staining for CD34 (**C**, H&E × 200; **D**, × 200). **(E, F)** The fibrosarcomatous component of DFSP (case 1) could exhibit loss for CD34 staining (**E,** H&E × 200; **F,** × 200).

Immunohistochemical analysis revealed that most of the tumors (57/66, 86.3%) were diffusely positive for CD34, whereas the minority (9/66,13.6%) showed patchy or negative staining. The decreased or lost expression of CD34 was mainly observed in myxoid and fibrosarcomatous areas, originating from three conventional DFSPs, three myxoid DFSPs, two FS-DFSPs, and one GCF (**Figure 2**). Majority of tumors were negative for smooth muscle actin (SMA), while there were a few cases (9/46, 20%) exhibiting positivity or focal staining. All cases were negative for desmin, S-100 protein, myogenin, Bcl-2, CD99, p16, p63, cytokeratin, and EMA.

### Molecular analysis

**Figure 3:**
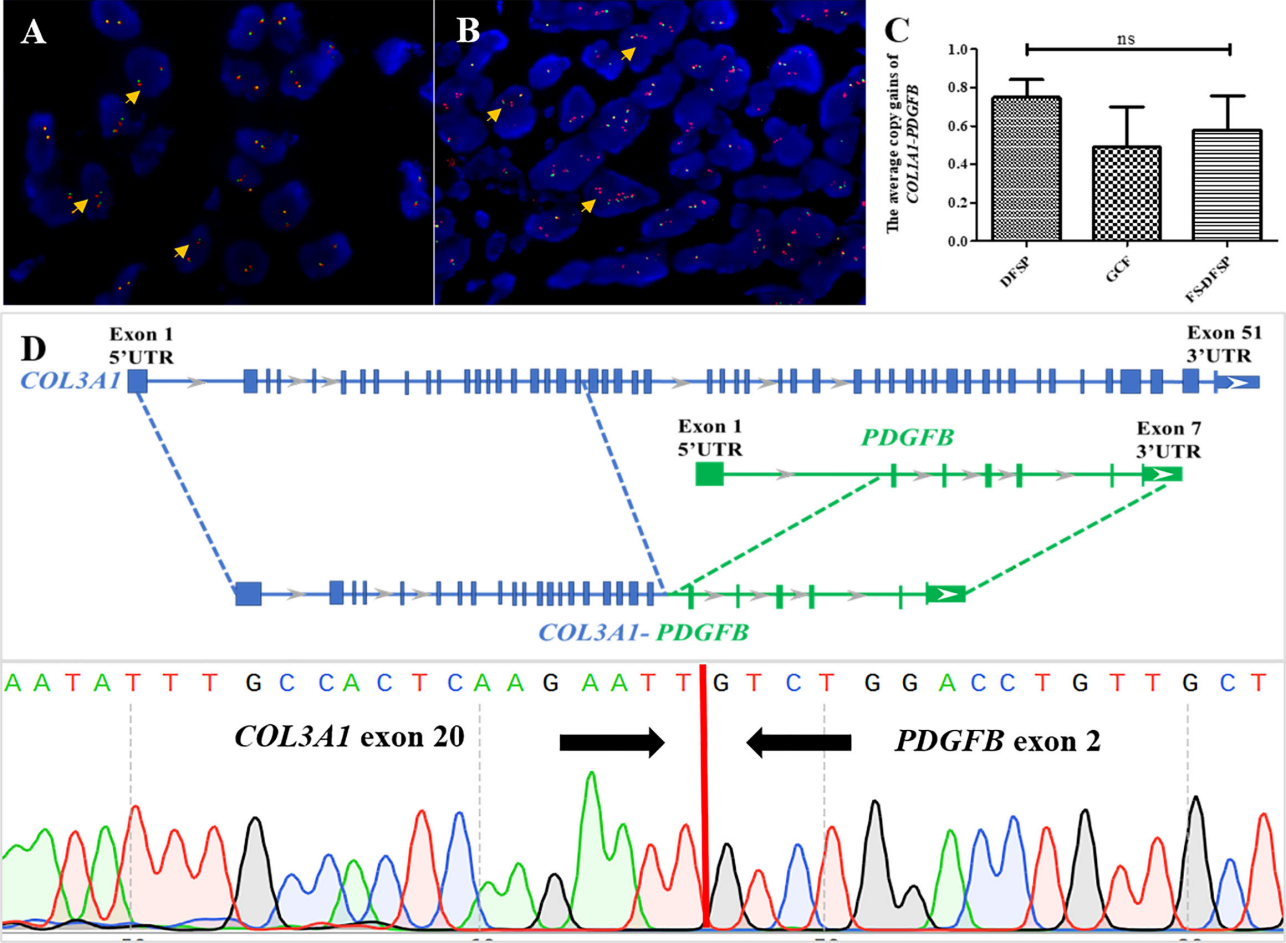
Molecular findings of pediatric DFSP. **(A)** FISH was performed on the DFSP component using a *PDGFB* break-apart probe to the locus on chromosome 22. The results showed several tumor cells with one red–green signal indicating a normal chromosome 22; one separate red and one separate green signal indicating a *COL1A1*-*PDGFB* fusion gene with no extra copies of red signal (orange arrows) (*PDGFB*, red signals and green signals). **(B)** FISH was performed on the DFSP component using a *PDGFB* break-apart probe to the locus on chromosome 22. The results showed several tumor cells with one red–green signal indicating a normal chromosome 22 and one to three extra copies of red signal (orange arrows) indicating a *COL1A1*-*PDGFB* fusion gene (*PDGFB*, red signals and green signals). **(C)** Comparison among DFSP (left column) and the GCF and the FS-DFSP (right two columns) indicated that there was not much difference in average *COL1A1*-*PDGFB* copy gains of pediatric DFSP. **(D)** Next-generation sequencing revealed case 65 with a novel *COL3A1* (e20)*-PDGFB* (e2) fusion (upper part); Sanger sequencing results demonstrated the presence of the *COL3A1-PDGFB* fusion gene (lower part).

FISH analysis indicated that 46 cases (46/53, 86.8%) were positive for *PDGFB* in a split-signal pattern. There were four that showed negative results of *PDGFB* rearrangement (cases 16, 23, 42, and 52), in one of which (case 16) *COL1A1*-*PDGFB* fusion was detected. One case exhibited an ambiguous result of *PDGFB* rearrangement (case 65). The last two cases failed the experiment (cases 5 and 13). The median *COL1A1*-*PDGFB* copy gain of pediatric DFSP was 0.7 (range 0–1.8; mean ± SD, 0.68 ± 0.46). Not much different from the classic DFSP, the median *COL1A1*-*PDGFB* copy gain in the FS subtype was 0.6 (range 0–1.1; mean ± SD, 0.58 ± 0.40) (*p* = 0.64), while in the GCF subtype, the median *COL1A1*-*PDGFB* copy gain was 0.45 (range 0–1.25; mean ± SD, 0.49 ± 0.50) (*p* = 0.36) (**Figures 3A–C**).

Moreover, 49 of 53 tested cases (92.5%) including all detected biopsy specimens (11/11, 100%) contained *COL1A1*-*PDGFB* fusion, while 4 cases were negative for *COL1A1*-*PDGFB* based on the routine FISH screening (cases 23, 42, 52, and 65). One case (case 65) with suspicious *PDGFB* split but *COL1A1*-*PDGFB* fusion negative was found to harbor a novel *COL3A1*-*PDGFB* fusion through NGS. The PCR and Sanger sequencing further confirmed the *COL3A1*-*PDGFB* fusion gene (**Figure 3D**).

### Treatment and follow-up

The treatment and follow-up information is detailed in **Table 1**. Surgical strategies for 63 patients were composed of marginal excisions (40/63, 63.5%) and WLE (23/63, 36.5%). Eight cases (8/15, 53.3%) underwent WLE after biopsies. One (case 14) underwent finger amputation for recurrent tumor therapy. Targeted therapy with imatinib mesylate was administered to one patient (case 7) after tumor excision on the second relapse.

Follow-up information was available on 49 patients (49/66, 74.2%) with a duration from 12 to 161 months (median, 60 months). Fourteen patients (14/49, 28.5%) developed recurrence, all with marginal excisions with positive margins. The others survived with no evidence of disease.

## Discussion

The occurrence of DFSP could involve people of all ages, from neonates to old adults (13, 20–22). Pediatric DFSPs were uncommon, which accounted for only 7.1% (66/926) of all DFSPs in our medical center. Among the DFSPs, the GCF subtype occurred mainly within 10 years old, which was consistent with previous observations as a pediatric-predominance subtype. The cohort of DFSP displayed a slight female predilection (M:F = 1:1.1), which was similar to the US pediatric population (11). Consistently, GCF occurred significantly in the male sex (M:F = 5:1) as the tendency of literature presenting male predominance in children diagnosed GCF (13, 14). The tumors in our cases were widespread and were predominantly distributed on the trunk (57.6%), followed by the extremities (30.3%) and head/neck (12.1%). Moreover, functional vitals or cosmetic positions including breast (4/66, 6%), groin (3/66, 4.5%), scalp (2/66, 3%), and face (1/66, 1.5%) could be involved and were rarely mentioned in children according to previous findings (12, 23, 24).

The distribution spectrum of the subtype exhibited some differences between pediatric and adult DFSPs. In the adult population, conventional DFSP was the most predominant subtype, which constituted approximately 61.3%–91% of DFSPs in previous studies, followed by FS-DFSP (10%–16%), myxoid DFSP (7.6%), pigmented DFSP (2.7%–5%), GCF (2.7%), and plaque-like DFSP (1.3%–1.7%) (3, 25–29). According to the results with detailed constitution of pediatric DFSP variants in this large cohort, the incidence of conventional DFSP (62.1%), FS-DFSP (9.1%), and myxoid DFSP (6.1%) was close to the lower limit of the adult counterparts, while pigmented DFSP (9.1%), GCF (9.1%), and plaque-like DFSP (4.5%) were higher than that of the adult.

CD34 is the most frequently used immunohistochemical marker for the diagnosis of DFSP. Typically, DFSP stains positive for CD34 in about 90% of tumors and mostly shows negativity for SMA (30, 31). In our series, 86.3% of cases were diffusely positive for CD34 expression, whereas diminished or absent staining presented in approximately 13.6% of pediatric DFSP cases. The decreased or lost expression of CD34 was mainly observed in myxoid and fibrosarcomatous areas, as published (3, 14, 32, 33). One case of classic DFSP showed a lack of CD34 expression (case 52), which two pathologists independently reviewed, arriving at a diagnosis of classic DFSP based on the typical histology. FISH utilizing the PDGFB break-apart probe revealed unbalanced translocation presenting additional 3’-red signals in 5% of these tumor cells, while the COL1A1-PDGFB fusion probe showed yellow signal denoting a fusion pattern in 2% of tumor cells. This case might show cryptic rearrangement associated with DFSP. Regretfully, the specimen could not be further investigated using NGS because of poor quality.

Evidence of *COL1A1*-*PDGFB* rearrangement is the key to the differential diagnosis of difficult cases and is inevitable for the effective application of targeted treatment. FISH has shown the validity for confirmation of the *COL1A1-PDGFB* fusion and it was widely applied to clinical detection. In our children’s series, 92.5% of children’s DFSPs were confirmed positive results by using the *PDGFB* break-apart probe and the *COL1A1*-*PDGFB* fusion probe as routine screening methods, similar to the previous FISH studies with detectable rate ranging from 86% to 96% (7, 8, 34). In addition, the fusion product *COL1A1*-*PDGFB* was amplified with low levels of the17q and 22q sequences (usually one to three copies), which could be detectable by either FISH or comparative genomic hybridization (GCH) (19, 35). In our study, we found that the average gains of the *COL1A1*-*PDGFB* fusion gene showed no statistical difference in ordinary DFSP, FS-DFSP, and GCF groups of children, among which the GCF cases presented the lowest average genomic gains. While correlated studies were scarce in children, more samples and deeper investigations are needed to further reveal the meaning of the molecular characters in pediatric patients.

Nevertheless, based on previous studies, a minority of DFSP cases with uncertain or negative results based on routine FISH assays are considered to be molecular unconfirmed DFSP and might result in inaccurate diagnosis (36). Application of supplementary NGS approaches would be of value to fusion detection (37). In our cohort, 7.5% (4/53) of pediatric DFSPs exhibited molecular unconfirmed characteristics. After carefully reviewing the published English articles, we included two studies and collected a total of seven pediatric DFSPs (including the four pediatric cases in our cohort) considered to be molecular unconfirmed based on routine FISH detection, the clinicopathological and molecular characteristics of which are summarized in **Table 2**. The clinicopathological features of most cases seem to not differ from the corresponding subtypes except one GCF (present case 65) with atypical morphology. Genetically important, NGS revealed the GCF containing a novel *COL3A1*-*PDGFB* fusion that was first presented in DFSP. *COL3A1*, located in 2q32.2, belongs to the collagen genes together with *COL1A1* and *COL1A2*, and encodes a structural protein of type III collagen, which is found in abundance in extensible connective tissues, such as skin, blood vessels, gastrointestinal tract, and the developing brain (38–41). The translocation of *COL3A1* had been reported as a rare partner fusing to *PLAG1*-rearranged neoplasms (including lipoblastoma and unclassified spindle cell neoplasm) and *USP6*-rearranged neoplasms (including cranial fasciitis, cellular fibroma of the tendon sheath, and unclassified benign myofibroblastic tumor). The breakpoints seem to constantly occur at exon 1 of *COL3A1* in *COL3A1*-*PLAG1* and *COL3A1*-*USP6* cases, whereas they occurred at exon 20 of *COL3A1* in the present GCF case (40, 42–46). In addition, atypical variants of *COL3A1* were associated with Ehlers–Danlos syndrome (EDS) involving connective tissue disorders (39, 47). Furthermore, though without unique clinical presentation, some distinctive pathological morphology was observed that could be associated with the *COL3A1*-rearranged GCF, which contained a higher proportion of multinucleated giant cells with larger and more atypical nuclei than the *COL1A1*-rearranged GCFs. However, more studies are needed to confirm the relationship between the specific chimerism and morphology. In addition, there was one classic DFSP with *COL6A3*-*PDGFD* and one FS-DFSP with *EMILIN2*-*PDGFD* in children from the work of Lee et al. (48). Dadone-Montaudié et al. described one pediatric pigmented DFSP, which was not found to have suspicious transcript even after using RNA sequencing, indicating that a more complicated mechanism might exist (36). There were three cases (present cases 23, 42, and 52) of genetic aberrations in the current study that could not be further identified using NGS because of the poor quality of the specimens, which could be associated with alternative rearrangement (including *PDGFD* rearrangement and *PDGFB* rearrangement with novel partner genes), cryptic *COL1A1*-*PDGFB* fusion, and even other sophisticated chromosomal aberrations (36, 49–52). We still needed more specimens to reveal the genetic characteristics of cryptic pediatric DFSP. Altogether, NGS could be a helpful strategy to identify the molecular unconfirmed DFSP and provide detailed information about abnormal genes for further investigation.

**Table 2:** Clinicopathologic and molecular characteristics of 7 cytogenetically cryptic DFSPs in children.

No.	Gender/Age	Location	Histologic findings	Diagnosis	CD34	FISH					RNA sequencing	PCR	Follow-up/ month
						COL1A1-PDGFB fusion	PDGFB break apart	PDGFD break apart	COL6A3 break apart	EMILIN2 break apart			
R1(48)	F/14	Neck	NA	DFSP	Positive	ND	Negative	Positive	Positive	NA	ND	NA	NA
R2(48)	F/15	Thigh	NA	FS-DFSP	Positive	NA	Negative	Positive	NA	Positive	ND	NA	NA
R3 (36)	M/4	Calf	Storiform pattern	Pigmented-DFSP	Positive	ND	Negative	Negative	Negative	Negative	No fusion transcript	NA	NED/17
Present case 23	M/16	Back	Myxoid matrix	Myxoid-DFSP	patchy	Negative	Negative	NA	NA	NA	NA	NA	R/10; NED/77
Present case 42	F/1	Hip	Pigmented cell, storiform pattern	Pigmented-DFSP	Positive	Negative	Negative	NA	NA	NA	NA	NA	NED/44
Present case 52	F/13	Forehead	Storiform pattern	Classic-DFSP	Negative	Negative	Negative	NA	NA	NA	NA	NA	NA
Present case 65	M/2	Forearm	Giant cell, myxoid matrix	GCF	partial	Negative	Ambiguous	NA	NA	NA	COL3A1(e20)-PDGFB(e2)	COL3A1(e20)-PDGFB(e2)	Recent case

ND, not detectable; NA, not available; R, relapse; NED, no evidence of disease.

The diagnosis of pediatric DFSP could be challenging, and it should be differentiated from not only benign mimics but also malignant neoplasms, similar to its subtypes in terms of the diversity of histological morphology in the pediatric tumor. Basically, the histologically and immunophenotypically (CD34 positive) overlapping pediatric lesions are often considered in the differential diagnoses, such as plaque-like CD34-positive dermal fibroma (lack of *COL1A1*-*PDGFB*), fibroblastic connective tissue nevus, fibrous hamartoma of infancy (EGFR exon 20 insertion/duplication mutations), pediatric NTRK-rearranged spindle cell neoplasm (co-expression of CD34 and S100, and NTRK-positive expression or rearrangement), lipofibromatosis, and plexiform myofibroblastoma. Although DFSPs are generally centered within the dermis or subcutis and characterized by spindle cells with a storiform to the whorled pattern, when these tumors have similar morphological features, the cytogenetic method can help with diagnosis, especially for small or superficial biopsy samples. In addition, there are a minority of DFSPs (especially myxoid DFSP and FS-DFSP) with patchy or negative staining for CD34 that are more likely to result in diagnostic pitfalls. The myxoid DFSP could be easily confused with other myxoid lesions such as superficial acral fibromyxoma, solitary fibrous tumor, and low-grade fibromyxoid sarcoma (LGFMS). Despite developing typically in old adults, pediatric liposarcoma should rarely be excluded, especially myxoid liposarcoma (ML) (53). FS-DFSP with atypical staining for CD34 could be confused with high-grade sarcomas, such as high-grade infantile fibrosarcoma (IFS) with *ETV6-NTRK3* fusion, especially dedifferentiated liposarcoma (DDL) without a well-differentiated liposarcoma (WDL) component. Notably, the situation we had reported could be extremely challenging due to the presence of *MDM2*/*FRS2* amplification and the lack of evidence for *COL1A1*-*PDGFB* fusion by routine FISH screening (54). Importantly, carefully looking for conventional components and searching for characteristics of fusion genes could be helpful for pathologists to confirm complicated cases.

Tissue biopsy, as one of the gold standard diagnostic examinations, could effectively assist in identifying uncertain lesions at an early stage. There were 15 (15/66, 22.7%) children who underwent biopsy, and 11 biopsy specimens detected by FISH all identified with *COL1A1*-*PDGFB* fusion, which reconfirmed the valuable function of FISH in biopsy samples as shown in our previous report (55). Therefore, it encouraged us to increase biopsy in children and boosted FISH application in pediatric specimens.

Accurate and early diagnosis is critical to guide an appropriate therapeutic scheme in children. Nowadays, the recommended treatment for DFSP is either WLE (2–4 cm) with tumor-negative margins or Mohs micrographic surgery (MMS) (56). In our group, 40 patients (63.5%) underwent marginal excisions, and the other 23 patients (36.5%) received WLE. The overall prognosis of pediatric DFSP was favorable, with all patients surviving without metastasis. However, 15 patients (15/49, 30.6%) developed tumor recurrence. It should be noted that there were 2 of 15 (13.3%) recurrent tumors transforming into fibrosarcomatous DFSP, which was more aggressive than the primary conventional types. Importantly, the recurrence of DFSP was closely related to surgical margin. A wide local excision (2–3 cm) with tumor-negative margins reduces the local recurrence of DFSP from 50%–75% to 0%–30% (24, 57). Moreover, MMS could further decrease the recurrence rate to 0.6%–6% (58). Noteworthy, a higher proportion of patients (8/15, 53.3%) were subjected to WLE, which was a benefit from early diagnosis through biopsy. It highlighted the pivotal role of biopsy in providing definitive evidence for patients to select an optimal therapeutic strategy. Moreover, 11 patients suffered defects from surgery involving anatomic critical regions including the breast, groin, finger, scalp, and face in this cohort. Actually, there are numerous issues arising from surgery for the special population: poor compliance of young patients, prolonged anesthesia duration, increasing surgical difficulty and risk, and unacceptable cosmetic and functional mutilation.

Another treatment opportunity that is typically reserved for adults in surgically unresectable, recurrent, or metastatic cases is targeted therapy (7). However, there is no standard clinical guidance about targeted therapy for pediatric patients. Hitherto, imatinib was applied on six child patients postoperatively for continuous remission and preoperatively for tumor reduction, all of whom achieved the desired effects (23, 59–62). In our study, we reported another patient (case 7) diagnosed with the GCF subtype who benefited from targeted therapy with imatinib after marginal excision in the second relapse, with no evidence of disease after 10 years. Therefore, using imatinib mesylate could act as a neoadjuvant or adjuvant therapy to help control tumor progression, in tandem with other treatments in children, but it still warrants further evaluation and large-scale investigation.

In conclusion, we present the largest series study of 66 cases of pediatric DFSP with genetic investigation. There are certain differences in clinicopathology between children and adults. Most pediatric DFSPs contain classical *COL1A1*-*PDGFB* fusion as compared with adults. The *COL3A1*-*PDGFB* chimerism might be associated with the special morphology of GCF, which needs further investigation. Furthermore, FISH screening, and even supplementary NGS detection, should be used in identifying pediatric lesions with typical or uncertain morphology involving dermis and subcutis. The overall prognosis would be favorable with appropriate treatment, while more attention should be paid to recurrence prevention and mutilation reduction in DFSP management in the special population.

## Data availability statement

The data presented in the study are deposited in the NCBI repository (https://www.ncbi.nlm.nih.gov/), accession number OQ262947.

## Author contributions

ZZ and YL analyzed the data and prepared the manuscript. CS collected the clinicopathological data of the patients. MC carried out the molecular studies. HZ, ZZ, XH and YL were responsible for diagnosis and review. HZ supervised and revised the manuscript. All authors contributed to the article and approved the submitted version.
